# Therapeutic Efficacy and Immunological Response of CCL5 Antagonists in Models of Contact Skin Reaction

**DOI:** 10.1371/journal.pone.0008725

**Published:** 2010-01-15

**Authors:** Miriam Canavese, Fiorella Altruda, Lorenzo Silengo

**Affiliations:** Department of Genetics, Biology and Biochemistry, University of Torino, Torino, Italy; University of Maryland School of Pharmacy, United States of America

## Abstract

Skin-infiltrating T-cells play a predominant role in allergic and inflammatory skin diseases such as atopic dermatitis, psoriasis and allergic contact dermatitis. These T-cells are attracted by several chemotactic factors including the chemokine CCL5/RANTES, a CC chemokine inducing both the migration and activation of specific leukocyte subsets. CCL5 has been found to be associated with various cell-mediated hypersensitive disorders such as psoriasis, atopic dermatitis and irritant contact dermatitis. We have used two antagonists, the first, Met-CCL5, a dual CCR1/CCR5 antagonist and the second, a variant in which GAG binding is abrogated, ^44^AANA^47^-CCL5, which acts as a dominant negative inhibitor of CCL5. The antagonists were tested in two models of contact skin reaction. The first, irritant contact dermatitis (ICD) is a pathological non-specific inflammatory skin condition arising from the release of pro-inflammatory cytokines by keratinocytes in response to haptens, usually chemicals. The second, contact hypersensitivity (CHS) is a T-cell dependent model, mimicking in part the T-cell-mediated skin diseases such as psoriasis. In both models, the CCL5 antagonists showed therapeutic efficacy by reducing swelling by 50% as well as the reduction of soluble mediators in homogenates derived from challenged ears. These results demonstrate that blocking the receptor or the ligand are both effective strategies to inhibit skin inflammation.

## Introduction

Chemokines are a large family of small structurally homologous cytokines that stimulate leukocyte movement and regulate migration of leukocytes from the blood to the tissue. Since the discovery of the super-family of chemokines and their receptors, there has been a considerable effort to define their particular role in the orchestration of leukocyte trafficking. Using a variety of experimental approaches, evidence has been provided that chemokines are essential mediators in the pathophysiology of inflammatory diseases and thus good candidates for therapeutic intervention strategies [1].

Chemokines play a pivotal role in cellular recruitment through interactions with both cell surface G protein-coupled receptors and glycosaminoglycans (GAGs) [2]. Specific GAG binding sites of several chemokines have been delineated by mutagenesis, demonstrating that these sites are either distinct, or partially overlap with receptor binding sites. For CCL5 the predominant binding site has been shown to be the BBXB motif in the 40s loop [3]. The variant [^44^AANA^47^]-CCL5, in which the three basic residues in this motif are mutated to alanine, loses 80% of its capacity to bind to the GAG heparin in vitro as compared with wild-type CCL5 [2], [3].

The recruitment of T cells and other leukocytes to the site of skin inflammation is a critical step for an efficient response to potentially dangerous signals as well as in the pathogenesis of chronic inflammatory skin diseases [1]. A hallmark of autoimmune skin diseases is the over-expression of chemokines resulting in a detrimental local accumulation of pro-inflammatory immune cells [2]. Cytokines and chemokines have a fundamental role in the regulation of leukocyte trafficking. The chemokine-chemokine receptor system is highly redundant and forms a complex network relevantly involved in the expression of inflammatory skin diseases, including irritant contact dermatitis, atopic dermatitis, allergic contact dermatitis and psoriasis. The pattern of chemokine expression shows overlapping features but also important differences in these diseases due to distinct sources and types of pro-inflammatory signals involved in chemokine induction and the inherent capacity of resident skin cells to produce chemokines. Various studies have documented a strong chemokine expression in psoriatic skin lesions [1], [4], [5], [6]. Specifically, CXCL8/IL-8 and the related CXCL2/Gro-β are strongly up-regulated in psoriatic skin and are responsible for the typical intra-epidermal collection of neutrophils. CCL2/MCP-1, and CCL5, attract predominantly monocytes as well as T cell subsets and CXCR3 ligands attract Th1 cells [1], [4], [7], [8].

The underlying pathogenesis involves three predominant and interdependent biologic processes: inflammation, epidermal hyperproliferation, and altered differentiation with parakeratosis. The homeostasis of the normal epidermis depends on a balance of growth regulatory signals, which are altered in psoriatic epidermis [9].

The aim of this study was to evaluate the therapeutic efficacy and the immunological response in irritant contact dermatitis (ICD) and contact hypersensitivity (CHS) mouse models of the antagonistic CCL5 mutants. ICD is a pathological non-specific inflammatory skin condition, arising from the response of pro-inflammatory cytokines by keratinocytes in response to haptens, usually chemicals [10], [11]. CHS is a T-cell-dependent model, mimicking T-cell mediated skin diseases, such as psoriasis.

It has been previously shown that Met-CCL5, an N-terminally modified human-CCL5 that inhibits against activity at two rodent chemokine receptors CCR1 and CCR5 [12] is effective in a number of disease models [13]. More recently [^44^AANA^47^]-CCL5 was shown to be a potent inhibitor of cellular recruitment confirmed by direct visualization of inhibition of cell rolling and adhesion using intravital microscopy [2]. The variant showed a mechanism of action based on disruption of GAG binding and oligomerization, that results in specific sequestration of CCL5 [2]. In order to demonstrate that blocking the receptor or the ligand are both effective strategies to inhibit skin inflammation, the variants were tested in the two mouse models of contact skin reaction (ICD and CHS), described above. These models were chosen because chemokines, in particular CCL5, facilitate direct communication between the innate and adaptive immune responses and are known to act as key mediators during the full development of the inflammatory response in skin diseases.

## Materials and Methods

### Animals

Balb/c female mice, 8–12 weeks of ages, were used to evaluate the efficacy and the immunological response of the [^44^AANA^47^]-CCL5 and Met-CCL5 in ICD and CHS mouse models.

Animals were obtained from Charles River Laboratory (Calco, Italy) and housed under constant environmental conditions. All in vivo studies were performed according to the European Council Directive 86/609/EEC and the Italian Ministry guidelines for care and use of experimental animals (decree # 116/92). All experimental protocols were authorized by the Italian Ministry of Health (decree # 111/2004 B).

### Reagents

Both variants [^44^AANA^47^]-CCL5 and Met-CCL5 were produced at the Merck-Serono Pharmaceutical Research Institute, Geneva, Switzerland, as previously described [2], [12]. Dexamethasone (Soldesam, pharmaceutical preparation), used as reference compound, was provided by Lab.Farmacologico, Milan, Italy.

The following haptens and irritant were used: 1-fluoro-2,4-dinitrobenzene (DNFB) (Sigma, St Louis, MO), 4-ethoxymethylene-2phenyl-2-oxazolin-5-one (Oxazolone) (Sigma, St Louis, MO), Croton oil (Fluka,Chemika, Switzerland).

Commercial human Myeloperoxidase (MPO) and 5-Bromo-2-Deoxyuridine (BrdU) were obtained from Sigma, St Louis, MO. For cytokines analyses, BD™ Cytometric Beads Assay (CBA) Mouse Inflammation Kit and Mouse Th_1_/Th_2_ Cytokine CBA Kit were used and obtained from Becton Dickinson, BD Biosciences/Pharmingen, San Diego, CA.

For immunostainings: Rabbit Polyclonal Antibodies against mouse keratin 6 (K6), mouse keratin 10 (K10) and mouse keratin 14 (K14) (Covance- Prodotti Gianni, Italy), Rabbit pAb IgG (code AB27478, Abcam); Polyclonal Rabbit Anti-human CD3 (code A0452, Dako), Negative Control Rabbit Immunoglobulin Fraction (Normal) (code X 0903, Dako), Vectastain ABC Elite Kit (Vector Laboratories, Burlingame, CA) and EnVison (Dako) were used.

### Experimental Protocol

#### Contact Hypersensitivity mouse model (CHS) response to Oxazolone

On day 0 mice were painted on the shaved back with 50 µL of Oxazolone 3% in acetone/olive oil (4∶1). At day 5 mice were challenged by applying 10 µL of Oxazolone 0.5% in acetone/olive oil (4∶1) to each side of the right ear. Dexamethasone was used as reference compound at 10 mg/kg. Therapy was applied 30 minutes after challenge by intra-peritoneal (i.p.) injection.

The specificity of CHS response is generally defined as the difference between ear swelling responses to a given hapten dose in naïve versus sensitized animals [14] mediated by T-cells. A baseline was done before the irritation. After the treatment, swelling was followed by measuring the ear thickness. Ear swelling was calculated as ((Tn – T5) right ear)-(Tn – T5) left t ear)), where Tn and T5 represent values of ear thickness at day n of investigation and day 5 prior to challenge, respectively.

In order to label proliferating cells, 24 hours before the sacrifice, 5-Bromo-2-Deoxyuridine (BrdU) (Sigma, St Louis, MO) 200 mg/kg single dose i.p. was injected.

After 24 hours mice were sacrificed, ear thickness was measured and ears were homogenized and used for extended analyses.

#### Contact Hypersensitivity mouse model response to DNFB

On day 0 mice were sensitized by applying 1-fluoro-2,4-dinitrobenzene (DNFB) (Sigma, St Louis, MO) 0.5% in Acetone∶oil (4∶1) on the shaved back. After 5 days the ear thickness of both ears at three points each was measured and mice were challenged with DNFB 0.2% and treated with [^44^AANA^47^]-CCL5/vehicle/dexamethasone, as reference compound at 0.5 mg/kg, 30 minutes after challenge via i.p. injection. 24 hours after challenge mice were sacrificed, ear thickness was measured and ear swelling was calculated as described above.

#### Irritant Contact Dermatitis mouse model (ICD)

The irritant contact dermatitis (ICD) model used was induced by applying a chemical irritant. On day 0 a 2% solution of Croton oil (Fluka,Chemika, Switzerland) in acetone/oil (5∶1) was applied to both surface of the right ear.

Mice were treated with the [^44^AANA^47^]-CCL5 or Met-CCL5/vehicle/dexamethasone, as reference compound at 0.5 mg/kg, 30 minutes after irritation via i.p. injection. The ear swelling was followed by measuring the ear thickness of both ears at three time points and calculating the mean per ear.

For all measurements a caliper (Mytutoyo, Urdorf, Switzerland) was used.

Six hours after irritation mice were sacrificed and an ear-punch/ear was taken for further immunological investigations.

#### Protein extraction

Frozen ears were homogenized, dissolved in Lysing Buffer (PBS 1X stored at 4°C, Na_3_VO_4_, NaF, NaPir) for 1 h and centrifuged at 13000 rpm, 4°C, for 15 minutes.

Supernatant was collected and protein quantification was done by Bradford method (Biorad). After quantification, 100 µg of extract of each sample were used to perform CBA. Dilutions, if needed, have been made in sample buffer (Biorad).

#### Myeloperoxidase (MPO) assay

One punch biopsy of challenged ears, 5 mm in diameter, was collected by using a skin punch. Ear biopsy was homogenized and extracted in Hexadecyltrimethylammonium bromide (HDMA) (Sigma, St Louis, MO) in 50 uM potassium phosphate buffer (pH 6.5) at a final concentration of 0.5%. MPO was evaluated by adding at 50 µL of sample, obtained as described above, 50 µL of 1% HDMA, 10 µL of o-dianisine at 1.25 mg/mL and 10 µL of hydrogen peroxide at 0.05%. The enzymatic reaction was stopped after 15 minutes and absorbance was measured at 450 nm.

#### Soluble markers detection

Ears were homogenized and a protein extraction was performed, as described above. Cytometric Beads Assay (CBA) Mouse Inflammation Kit (Becton Dickinson, BD Biosciences/Pharmingen, San Diego, CA) was used to quantitatively measure Interleukin-6 (IL-6), Interleukin-10 (IL-10), Monocyte Chemoattractant Protein-1 (MCP-1), Interferon-γ (IFN-γ), Tumor Necrosis Factor (TNF) and Interleukin-12p70 (IL-12p70) protein levels in a single sample. As described, Mouse Th_1_/Th_2_ Cytokine CBA Kit (BD) was used to measure Interleukin-2 (IL-2), Interleukin-4 (IL-4), Interleukin-5 (IL-5), Interferon-γ (IFN-γ) and Tumor Necrosis Factor-α (TNF-α) protein levels in a single sample to identify a T-cell mediated immunological profile.

CBA data were analyzed with BD CBA Software, following manufacture's instructions. Detection limit of CBA assay is 20 pg/mL.

#### Tissue processing and immunostaining

Ears were collected, embedded in paraffin and the fixed sections were immunostained.

Immunohistochemical staining of keratynocyte proliferation and differentiation markers (K6 and K10) and CD3 staining were performed following manufacture's instructions. Rabbit polyclonal antibodies against mouse keratin 6 (K6), mouse keratin 10 (K10) and mouse keratin 14 (K14) (Covance- Prodotti Gianni, Italy), Rabbit pAb IgG (code AB27478, Abcam); Polyclonal Rabbit Anti-human CD3 (code A0452, Dako), Negative Control Rabbit Immunoglobulin Fraction (Normal) (code X 0903, Dako) were used and the staining was revealed with Vectastain ABC Elite Kit (Vector Laboratories, Burlingame, CA) and EnVision (Dako).

#### Histology

Standard protocol for Hematoxilyn-Eosin (H&E) staining was used.

#### Statistical analysis

GraphPad Prism4 (San Diego,CA) was used to perform statistical analyses.

Analysis of variance (ANOVA) followed by Dunnet or Bonferroni's post-tests was used. Data were expressed as mean ± standard error of the mean (SEM) and levels of significance were assigned as follows: *p<0.05, **p<0.01, ***p<0.001.

## Results

### Ear Swelling in Oxazolone-Induced CHS

**Figure 1:**
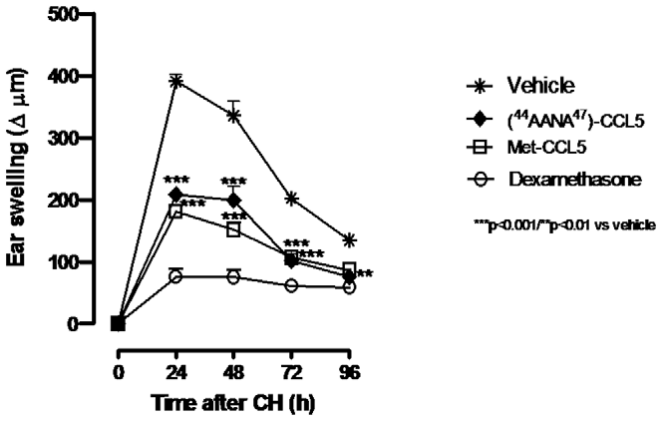
Therapeutic efficacy of [^44^AANA^47^]-CCL5 and Met-CCL5 on ear swelling. Ear Swelling in head to head Oxazolone-induced CHS. CHS was induced by sensitization on shaved back with 3% Oxazolone at day 0. Challenge with 0.5% Oxazolone was done at day 5 to the right ear of sensitized mice. Mice were treated 30′ after irritation once with CCL5 antagonists at 0.5 mg/kg ip. Dexamethasone 10 mg/kg was used as reference compound. Results are given as mean ± SEM of n = 8 mice per group. All statistical analyses were performed using two-way ANOVA followed by Bonferroni's post test (***p<0.001 vs vehicle for each time point- 24,48,72 h).

As shown in Fig. 1 the two variants were tested at 0.5 mg/kg in a head to head comparison CHS time course, using Oxazolone as hapten. A similar therapeutic potency was observed for the two compounds. 24 hours after challenge Met-CCL5 at 0.5 mg/kg was slightly more effective than [^44^AANA^47^]-CCL5 in terms of percentage of swelling reduction (54% vs 47%), but statistically the two variants showed a comparable therapeutic efficacy.

**Figure 2:**
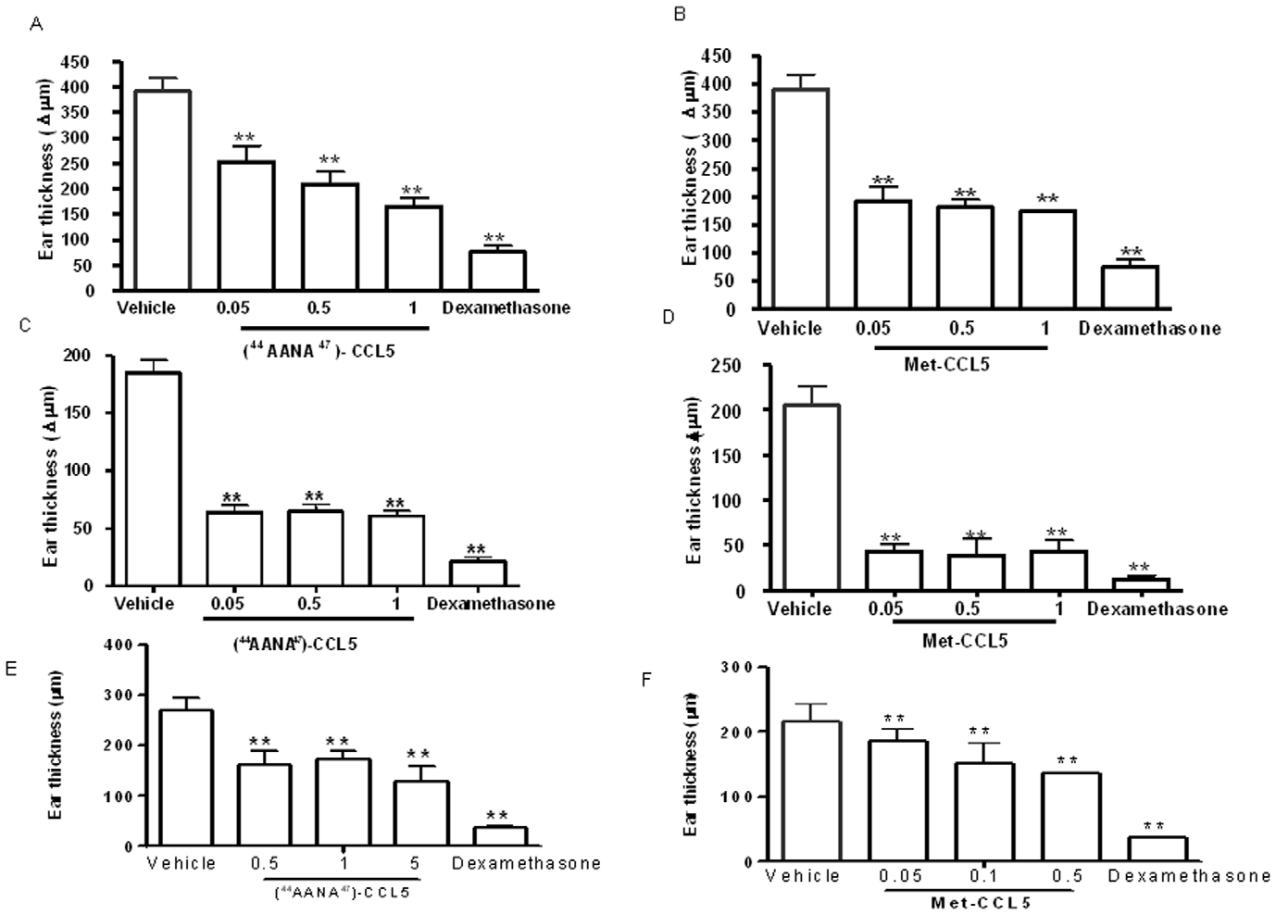
Therapeutic efficacy of [^44^AANA^47^]-CCL5 and Met-CCL5 on ear swelling, using different mouse models of contact skin reaction. **A**) represents [^44^AANA^47^]-CCL5 and **B**) Met-CCL5 therapeutic effect respectively on swelling in Oxazolone-induced CHS mouse model at 0.05–0.5–1 mg/kg dose via i.p. Dexamethasone 10 mg/kg sub-cute (s.c.) was used as reference compound. **C**) represents [^44^AANA^47^]-CCL5 and **D**) Met-CCL5 therapeutic effect respectively on swelling in CHS mouse model using DNFB as hapten at 0.05–0.5–1 mg/kg via i.p. Dexamethasone 0.5 mg/kg s.c. was used as reference compound. **E–F**) are showing therapeutic efficacy of [^44^AANA^47^]-CCL5 and Met-CCL5 in ICD mouse model tested at 0.5–1–5 mg/kg and 0.05–0.1–0.5 mg/kg i.p respectively. Dexamethasone 0.5 mg/kg was used as reference compound. All data were expressed as mean ± SEM of n = 8/group. All statistical analyses were performed using one-way Anova followed by Dunnett's multiple comparison post test (*p<0.05, **p<0.01, ***p<0.001 vs saline-treated vehicle group).

A pronounced ear swelling was observed in the control group (vehicle), elicited by hapten application 5 days after sensitization. Fig. 2A) and Fig. 2B) respectively represent the therapeutic effect on ear swelling of [^44^AANA^47^]-CCL5 and Met-CCL5 tested at 0.05–0.5–1 mg/kg via i.p.

Dexamethasone 10 mg/kg s.c. was used as reference compound. [^44^AANA^47^]-CCL5 was able to decrease significantly ear swelling and a bell shaped dose response was detectable with the increase of the doses from 1 mg/kg up to 10 mg/kg (data not shown). Met-CCL5 has shown high activity in decreasing ear swelling in a dose response modulation.

### Ear Swelling in DNFB-Induced CHS

Swelling, as major read-out, was followed after challenge by measuring ear thickness.

Treatments took place 30′ after challenge by intraperitoneal route at dose of 0.05–0.5–1 mg/kg.

The [^44^AANA^47^]-CCL5 and Met-CCL5 variants were able to significantly reduce the swelling 24 h post challenge. Met-CCL5 was slightly more active than [^44^AANA^47^]-CCL5 at 0.5 mg/kg (Fig. 2C–D).

### Ear Swelling in Irritant Contact Dermatitis (ICD) Mouse Model

Ear swelling, followed by measuring ear thickness, after hapten irritation was used as main read-out.

Animals were treated with [^44^AANA^47^]-CCL5 at doses of 0.5–1–5 mg/kg. The compound was able to significantly reduce the swelling, starting from 0.5 mg/kg and the maximum percentage of reduction was reached at 5 mg/kg. (Fig. 2E). Also treatment with Met-CCL5 was able to significantly reduce the swelling as shown in Fig. 2F.

### Myeloperoxidase Activity in ICD and CHS Ear Extracts

In ICD mouse model [^44^AANA^47^]-CCL5 at 1 and 5 mg/kg decreased significantly MPO activity to a similar level as Dexamethasone which was used as reference compound. The maximum percentage of reduction was 72.24% (27.76% of vehicle) at 1 and 5 mg/kg.

**Figure 3:**
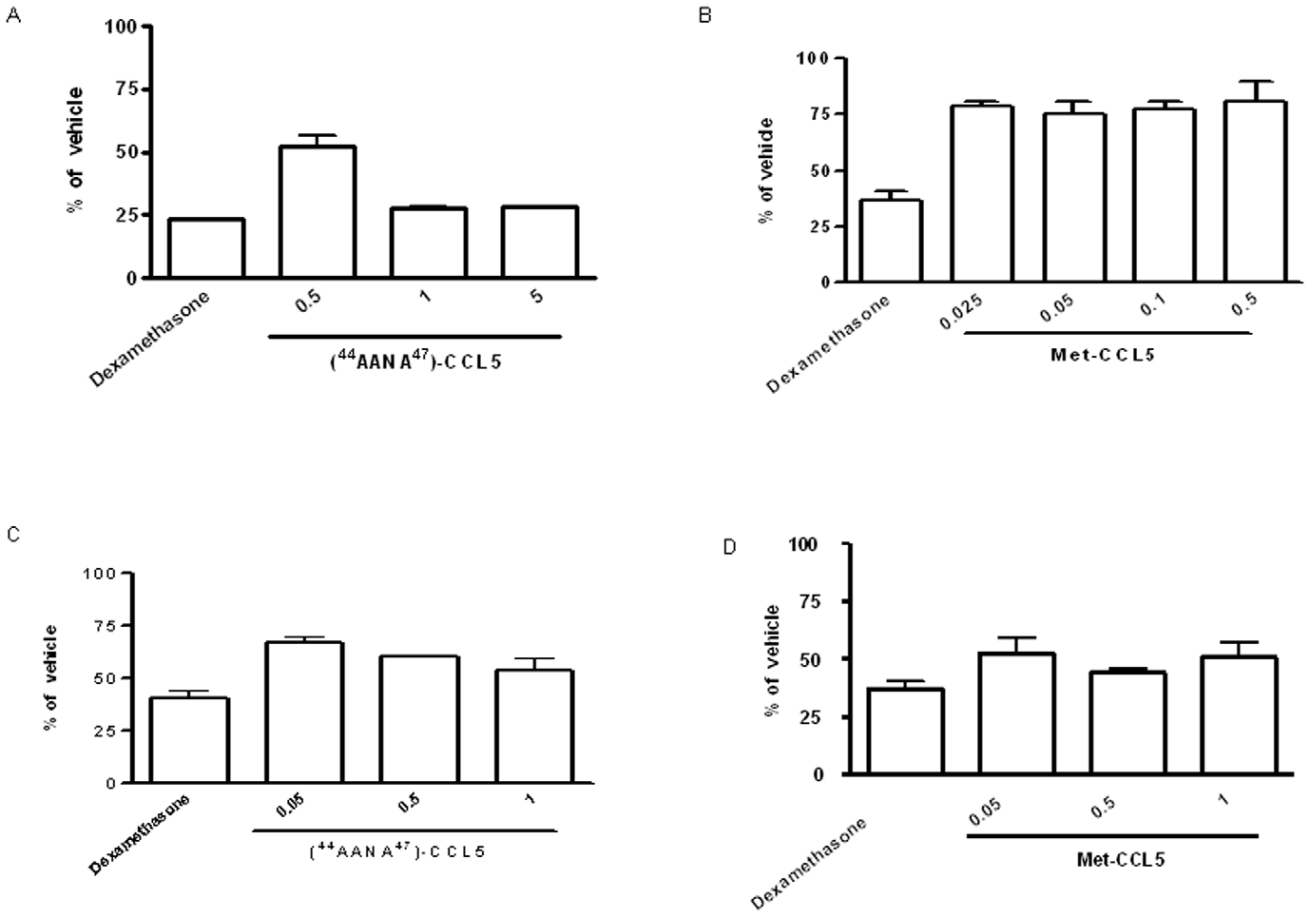
Myeloperoxidase activity in ICD and CHS ear extracts. Determination of MPO was used as an indirect measure of neutrophils recruitment and content of ear tissue. Myeloperoxidase activity was tested respectively in ICD and CHS mouse models. In ICD mouse model, one biopsy of challenged ear (5 mm of diameter) of balb/c 8–12 weeks females was irritated with Croton oil and treated after irritation with **A**) [^44^AANA^47^]-CCL5 at 0.5–1–5 mg/kg and **B**) Met-CCL5 at 0.025–0.05–0.1–0.5 mg/kg via intraperitoneal injection. In DNFB-induced CHS (Fig. **C/D**) one biopsy of challenged ear (5 mm of diameter) of balb/c 8–12 weeks females was challenged by 0.2% DNFB and treated 30′ after irritation with [^44^AANA^47^]-CCL5 and Met-CCL5 at 0.05–0.5–0.1 mg/kg via intra-peritoneal injection. Results are given in % of vehicle for comparison of therapeutic efficacy of the two mutants, as mean ± SEM of n = 4 mice.

Met-CCL5 was able to decrease MPO activity, but not in a dose dependent manner (Fig. 3A and Fig. 3B respectively).

An important reduction of MPO activity was observed in a T-cells dependent model of skin inflammation (DNFB-induced CHS) by [^44^AANA^47^]-CCL5 at 1 mg/kg (46.6% (53.4% of vehicle) (Fig. 3C).

As well as treatment with Met-CCL5 at 0.5 mg/kg and 1 mg/kg showed a maximum percentage of reduction close to 50% (44.20% of vehicle and 50.58% of vehicle respectively) (Fig. 3D).

### Cytokine and Chemokine Profiles in ICD and CHS Ear Extracts

**Figure 4:**
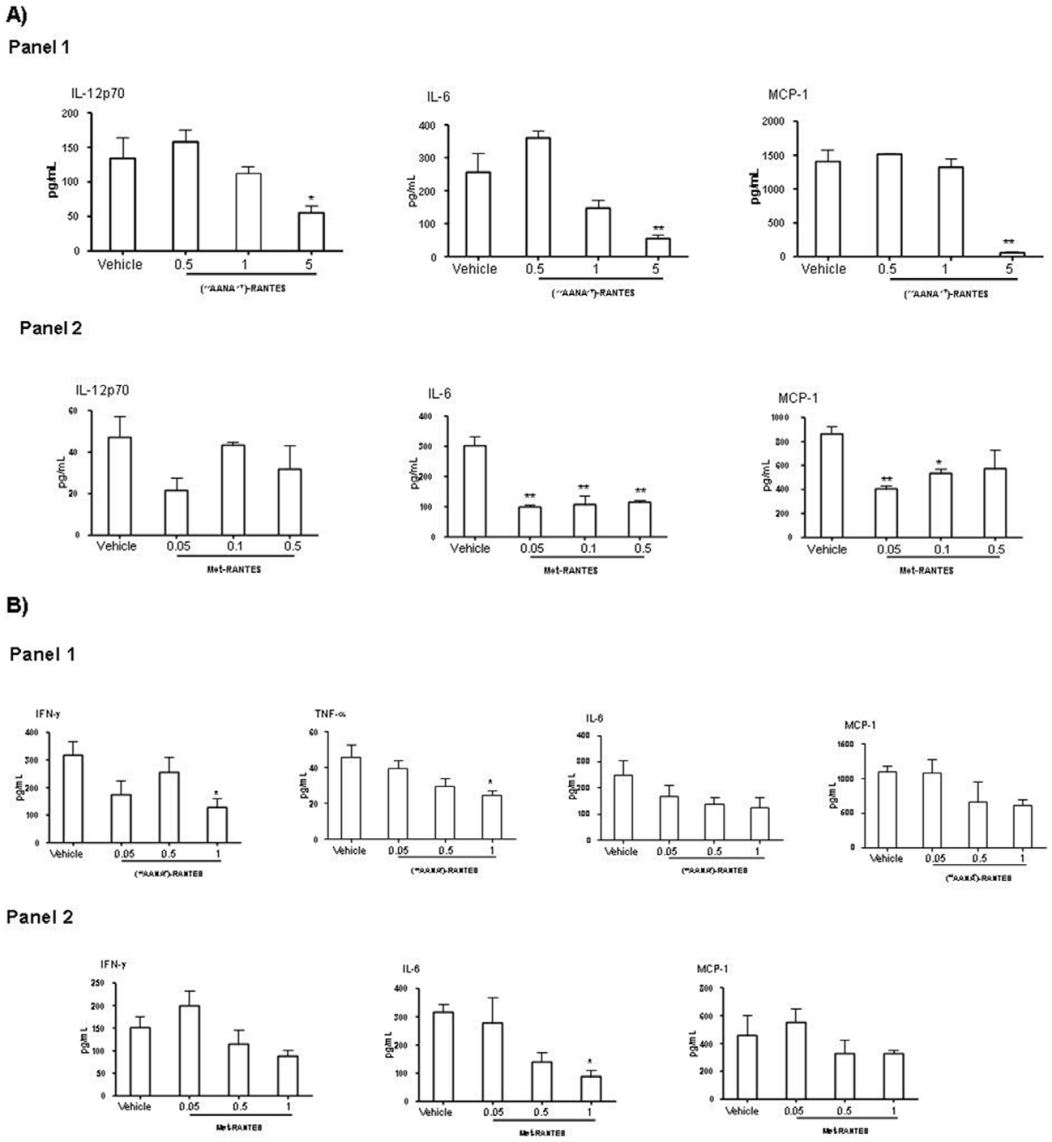
Cytokines and Chemokines profile in ICD and CHS ear extract. **A**) Cytokine and Chemokine profiles in ICD mouse model. CBA assay was performed on ear extracts of Balb/c female 8–12 weeks of age. **Panel 1**: Treatments used are represented by [^44^AANA^47^]- CCL5 at 0.5–1–5 mg/kg ip. [^44^AANA^47^]- CCL5 at 5 mg/kg was able to reduce significantly levels of IL-12p70 (*p<0.05), IL-6 (**p<0.01) and MCP-1 (**p<0.01). A trend to reduction has been observed for IL-6 and MCP-1 at 1 mg/kg dose. **Panel 2**: Treatments used are represented by Met-CCL5 at 0.05–0.1–0.5 ip. Met-CCL5 at 0.05–0.1–0.5 mg/kg was able to significantly reduce IL-6 (**p<0.01) and MCP-1 (**p<0.01, *p<0.05). A trend to reduction is shown for IL-12p70. Results are given as mean ± SEM of n = 4 mice per group. All statistical analyses were performed using one-way ANOVA followed by Dunnett's multiple comparison test (*p<0.05, **p<0.01 vs vehicle). **B**) Cytokine and Chemokine profiles in DNFB-induced CHS. CBA assay was performed on ear extracts of sensitized and challenged Balb/c female 8–12 weeks of age. **Panel 1**: Treatments used are represented by [^44^AANA^47^]-CCL5 at 0.05–0.5–1 mg/kg ip. [^44^AANA^47^]- CCL5 at 1 mg/kg significantly down-modulated IFN-γ and TNF-a (*p<0.05). A trend to reduction for IL-6 and MCP-1. **Panel 2**: Treatments used are represented by Met- CCL5 at 0.05–0.5–1 mg/kg ip. Met- CCL5 at 1 mg/kg significantly reduced IL-6 (*p<0.05). Trend to reduction was observed for IFNγ and MCP-1. Results are given as mean ± SEM of n = 4 mice per group. All statistical analyses were performed using one-way ANOVA followed by Dunnett's multiple comparison post test (*p<0.05).

Modulation of pro-inflammatory cytokines in ICD mouse model is shown in Figure 4A.

CBA assay was performed on ear extracts of Balb/c female 8–12 weeks of age.

Treatments are represented by [^44^AANA^47^]- CCL5 at 0.5–1–5 mg/kg, Met-CCL5 at 0.05–0.1–0.5 i.p.

[^44^AANA^47^]- CCL5 at 5 mg/kg was able to reduce significantly levels of IL-12p70 (*p<0.05), IL-6 (**p<0.01) and MCP-1 (**p<0.01).

A trend to reduction has been observed for IL-6 and MCP-1 at 1 mg/kg dose. TNF-a was below detection limit (20 pg/mL) (Fig. 4A panel 1).

Treatment with Met-CCL5 at 0.05–0.1–0.5 mg/kg was able to significantly reduce IL-6 (**p<0.01) and MCP-1 (**p<0.01, *p<0.05). A trend to reduction is shown for IL-12p70. TNF-a was below detection limit (20 pg/mL) (Fig. 4A panel 2).

Results are given as mean ± SEM of n = 4 mice per group. All statistical analyses were performed using one-way ANOVA followed by Dunnett's multiple comparison test (*p<0.05, **p<0.01 vs vehicle).

Figure 4B shows the analysis of cytokine levels in DNFB-induced CHS.

CBA assay was performed on ear extracts of sensitized and challenged Balb/c female 8–12 weeks of age. Treatments used are represented by [^44^AANA^47^]- CCL5 and Met-CCL5 at 0.05–0.5–1 mg/kg ip.

[^44^AANA^47^]- CCL5 at 1 mg/kg significantly down-modulated IFN-g and TNF-a (*p<0.05). A trend to reduction for IL-6 and MCP-1 was observed. Il-12p70 was below detection limit (20 pg/mL) (Fig. 4B panel 1).

Met- CCL5 at 1 mg/kg significantly reduced IL-6 (*p<0.05). Trend to reduction was observed for IFNγ and MCP-1. TNF-a and IL-12p70 were under detection limit (20 pg/mL) (Fig. 4B panel 2).

Results are given as mean ± SEM of n = 4 mice per group. All statistical analyses were performed using one-way ANOVA followed by Dunnett's multiple comparison post test (*p<0.05).

### Evaluation of Infiltrating Inflammatory Cells in DNFB-Induced CHS

**Figure 5:**
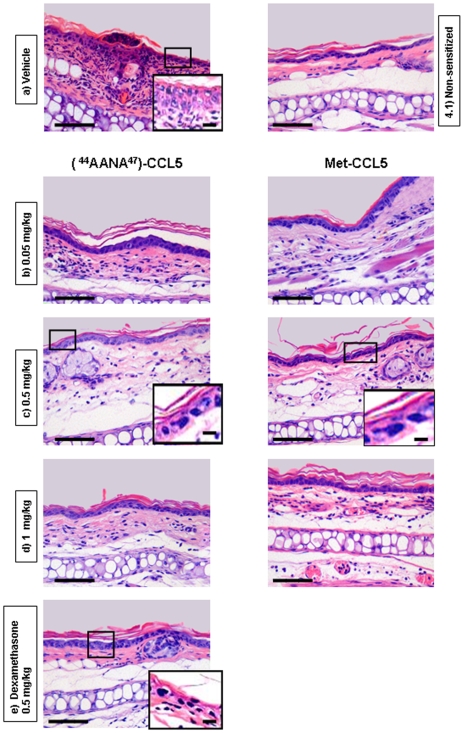
Evaluation of infiltrating inflammatory cells in ear sections. Haematoxylin and Eosin staining on paraffin ear sections of Balb/c females 8–12 weeks of age sensitized and challenged in a DNFB-induced CHS. 30′ after challenge, mice were treated with [^44^AANA^47^]-CCL5 and Met-CCL5 at 0.05–0.5–1 mg/kg ip. Dexamethasone 0.5 mg/kg sc was used as reference compound. **4a**) Vehicle group: high level of infiltration, polymorphonuclear cells (PMN) mostly, dendritic cells (DCs) and Natural Killer cells (NK) as lowest populations. A detail of hyperplasia of epithelium, represented by an increase in keratinocyte layer thickness is also shown. **4b**) Cellular infiltrates and hyperplasia of epithelium are observed in ear sections of animals treated with [^44^AANA^47^]-CCL5 and Met-CCL5 at 0.05 mg/kg. **4c**) [^44^AANA^47^]-CCL5 and Met-CCL5 at 0.5 mg/kg were able to decrease cellular infiltrates, hyperplasia of epithelium (detail shown in the panel) and dermis size (edema) to similar level as Dexamethasone. **4d**) Reduction of cellular infiltrates and keratinocyte layers thickness is observed for [^44^AANA^47^]-CCL5 and Met-CCL5 at 1 mg/kg. **4e**) Dexamethasone 0.5 mg/kg was used as reference compound. **4.1**) Control group: not-sensitized, normal skin conditions. In all panels, magnification 20× ( scale bar: 16.5 µm), details 40× (scale bar: 20 µm).

Hematoxilyn and Eosin staining was performed on ear sections 24 h post challenge (Fig. 5). The panel representing the vehicle (Fig. 5a) shows a massive infiltration and an increase in keratinocyte layer thickness pointing to hyperplasia of epithelium, a hallmark of psoriasis. Presence of cellular infiltrates was observed for the two variants tested at 0.05 mg/kg (Fig. 5b). As shown in Fig. 5 panels c,d [^44^AANA^47^]-CCL5 and Met-CCL5 at doses of 0.5 mg/kg and 1 mg/kg, with comparable efficacy, were able to decrease hyperplasia of epithelium, edema and cellular infiltration to a similar level as Dexamethasone, used as reference compound (Fig. 5e).

### Evaluation of T-Cells Recruitment in Oxazolone-Induced CHS

**Figure 6:**
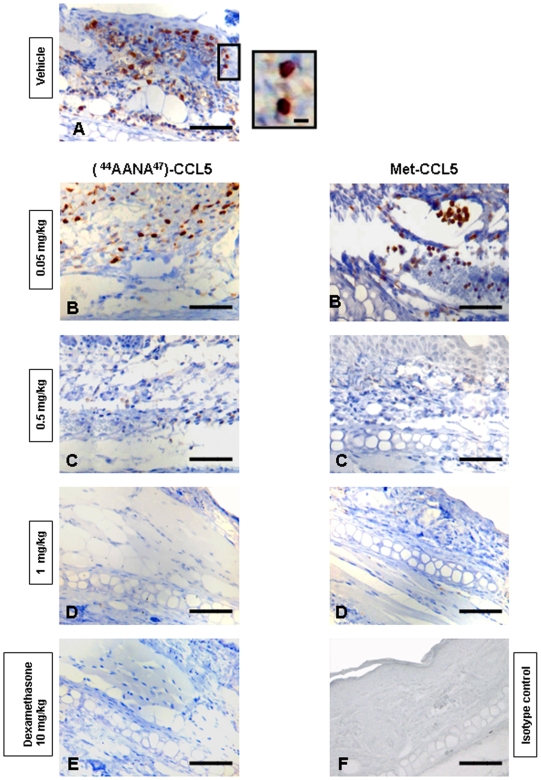
Evaluation of CD3 infiltrates in ear sections. Immunostaining with CD3 antibody on ear (paraffin sections) of Balb/c females 8–12 weeks of age sensitized and challenged in a Oxazolone-induced CHS. In all panels, magnification 40× (scale bar: 20 µm). **A**) Vehicle group: the CD3 staining on ear section revealed an important T cells involvement. **B**) No reduction of T cell infiltrates in ear sections is observed for [^44^AANA^47^]-CCL5 and Met-CCL5 at 0.05 mg/kg. **C–D**) [^44^AANA^47^]-CCL5 and Met-CCL5 at 0.5 and 1 mg/kg respectively were able to reduce T cell involvement to similar level as Dexamethasone. **E**) Dexamethasone 10 mg/kg was used as reference compound: clearly no staining is observed. **F**) Isotype control.

Figure 6 is representing CD3 infiltrates in ear sections in a Oxazolone-induced CHS. The staining of the vehicle group revealed an important involvement of T cells (Fig. 6a). [^44^AANA^47^]-CCL5 and Met-CCL5 at 0.05 mg/kg (Fig. 6b) were not able to reduce T cell recruitment. Both variants at 0.5 and 1 mg/kg reduced T-cells infiltrates to similar level as Dexamethasone (Fig. 6c/d). The staining revealed no presence of T cells in ear tissue of animals treated with Dexamethasone 10 mg/kg (Fig. 6e). Isotype control is shown in Fig. 6f.

### Evaluation of Epidermal Alterations in Oxazolone-Induced CHS

**Figure 7:**
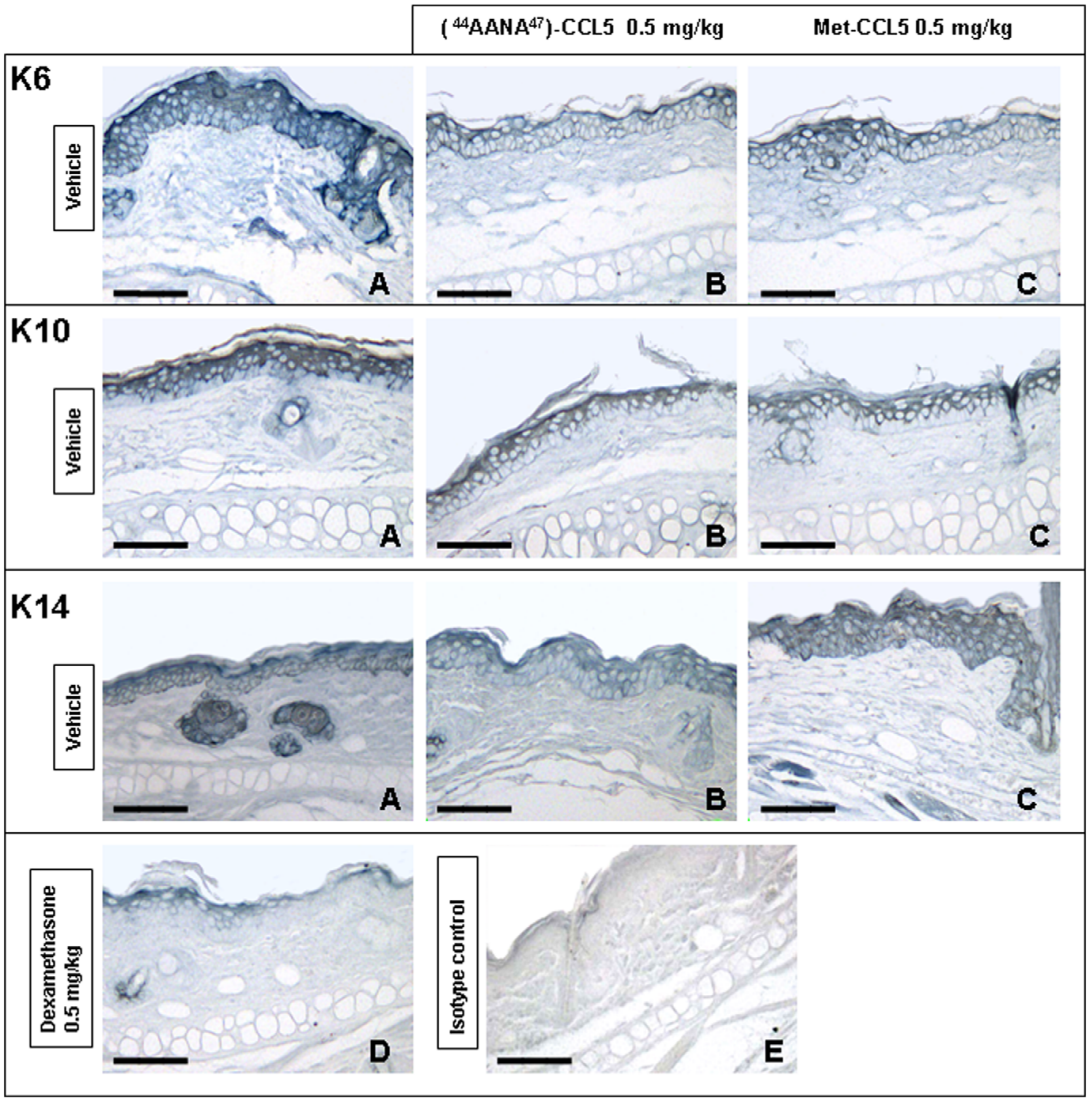
Expression of keratinocyte proliferation and differentiation markers in ear sections. Immunostaining for keratins was performed on paraffin sections of ear skin of Balb/c females 8–12 weeks of age sensitized and challenged by Oxazolone. Treatments with [^44^AANA^47^]-CCL5 and Met-CCL5 0.5 mg/kg via i.p. took place 30′ post challenge. Dexamethasone 10 mg/kg sc was used as reference compound. **A**) Vehicle: important hyperproliferation of keratinocyte layer: K6 is widely expressed and it is found supra-basal, K14 is constitutively expressed. K10 showed an inverse staining compared to K6. **B–C**) [^44^AANA^47^]-CCL5 and Met-CCL5 0.5 mg/kg respectively were able to down-modulate K6 and K10 expression and to reduce hyperproliferation in a similar manner. K14 staining showed no changes. **D**) Dexamethasone 10 mg/kg used as reference compound: clearly no staining is observed. **E**) Isotype control. In all panels, magnification 20× (scale bar: 16.5 µm).

A representative picture of the expression of K6, K10 and K14 is depicted in Figure 7. Using an Oxazolone-induced CHS, in the present study, we have investigated on keratinocyte proliferation/differentiation. Fig. 7 panel A shows an important hyperproliferation and differentiation of keratinocytes in the vehicle group. The two antagonists at 0.5 mg/kg were able to down-modulate K6 and K10 expression with a similar potency as shown in Fig. 7 panels B and C. K14 is constitutively express in basal cells and K14 staining showed no changes. No hyperproliferation is observed in ear tissue of animals treated with Dexamethasone (Fig. 7 panel D). Isotype control is shown if Fig. 7 panel E.

## Discussion

A hallmark of inflammatory skin disorders is a massive recruitment of leukocytes to the site of inflammation. Leukocyte recruitment is a well-orchestrated process that involves several protein families, including pro-inflammatory cytokines, adhesion molecules and chemokines [15].

Recent studies have provided evidence that chemokines are essential mediators in the pathophysiology of inflammatory diseases, and are thus good candidates for therapeutic strategies [1].

Modified chemokine peptides have emerged as useful and convenient tools for examining effects of chemokine receptor blockade [16].

In this study we tested two CCL5 antagonists in two models of contact skin reaction, ICD and CHS, to demonstrate that blocking the receptor or the ligand are both effective strategies to inhibit skin inflammation.

In 1997 Texeira and colleagues described a novel mouse model of eosinophil recruitment in which it was compared the in vivo chemoattractant activity of different CC chemokines. Their results demonstrate that Met-CCL5 acts on the mEotaxin receptor CCR3. When administered systematically, Met-CCL5 inhibited eosinophil recruitment into sites of allergic inflammation in mouse skin by 68% [17].

These data are consistent with our findings, although we used two different models of contact skin reaction. In fact, both in ICD, that results in activation of innate immune response with infiltration in the ear of mainly neutrophils and macrophages, and in CHS, which is instead a T cell dependent model of skin inflammation, Met-CCL5 showed a therapeutic efficacy by reducing swelling by 50%.

Myeloperoxidase (MPO) is an enzyme characteristic for granulocytes, playing major role in the metabolic activity of neutrophils [18], localized in the intracellular granules of neutrophils [19]. We used determination of MPO activity as an indirect measure of neutrophils recruitment and content of ear tissue, since the enzymatic activity correlates with the number and activation state of polymorphonuclear cells (PMN). We observed in both models reduction of MPO activity by Met-CCL5.

Down-regulation of IL-12p70, IL-6, MCP-1 and IFN-g by Met-CCL5 in both models of contact skin reaction suggest efficacy of the compound in modulation of both innate and adaptive responses.

These results were confirmed by histology and immunohistochemistry.

Hematoxilyn and Eosin staining and epidermal analyses using hyperproliferation and differentiation markers revealed Met-CCL5 efficacy at 0.5 and 1 mg/kg in decreasing cellular infiltration and epidermal hyperplasia in ear section as well as CD3 staining suggested that the compound at 0.5 and 1 mg/kg was able to modulate adaptive immune responses.

Moreover, it has been published by Elsner et al. that Met-CCL5 demonstrated significant inhibitory effects in acute and chronic models of tissue inflammation in vivo, reduction of leukocytes infiltration into affected tissues and an important efficacy in blocking chemokine-induced effector functions of human eosinophils in vitro [20]. It was observed that Met-CCL5 could modify the composition of leukocyte infiltrates by selective blockade of CCR1 and CCR5, but it has been shown that blocking chemokine - chemokine receptor interactions with Met-CCL5 was largely ineffective in EAE [11].

Recently, it was demonstrated that a CCL5 variant deficient in GAG binding, [^44^AANA^47^]-CCL5, antagonizes CCL5-induced recruitment. This antagonistic property was translated into an anti-inflammatory effect in a murine model of multiple sclerosis (MS), experimental autoimmune encephalomyelitis (EAE), in which its administration before disease onset significantly reduced clinical symptoms [21].

In our study the antagonistic property of [^44^AANA^47^]-CCL5 was extended into an anti-inflammatory effect in two murine models of contact skin reaction.

Swelling was used as indication of edema and subsequent skin reaction. [^44^AANA^47^]-CCL5 at 0.5 mg/kg was able to significantly reduce ear swelling in Oxazolone/DNFB-induced CHS which was confirmed by a bell shaped dose response detected with the increase of the doses from 1 to 10 mg/kg in a Oxazolone-induced CHS. Compound efficacy has been observed also in ICD mouse model. The maximum percentage of reduction was reached at 5 mg/kg.

Neutrophils recruitment was modulated by the compound at 1 and 5 mg/kg in innate and adaptive immune responses. This was suggested by reduction of MPO activity (72.24% in ICD and 46.6% in CHS mouse models). The compound was able to down-regulate pro-inflammatory cytokines. IL-12p70 and IL-6 were down-modulated in innate responses. As well as [^44^AANA^47^]-CCL5 at 0.5 and 1 mg/kg was able to reduce IFNγ, IL-6 and MCP-1 in adaptive immune responses. Histology and immunohistochemistry confirmed compound efficacy at 0.5 and 1 mg/kg in modulation of innate and adaptive immunity in mouse models of contact skin reaction. [^44^AANA^47^]-CCL5 at 0.5 and 1 mg/kg, with comparable efficacy to Met-CCL5 at same doses, was able to decrease hyperplasia of epithelium, edema and cellular infiltration to a similar level as reference compound (Dexamethasone 0.5 mg/kg) As well as CD3 staining revealed an important reduction of T-cells recruitment in ear tissue of animals treated with [^44^AANA^47^]-CCL5 at 0.5 and 1 mg/kg in CHS, which indicate a modulation of adaptive immunity.

These data taken together suggest that both compounds, taking part with different mechanisms of action, showed therapeutic efficacy at 0.5 and 1 mg/kg in modulating innate and adaptive immune responses in mouse models of skin reaction.

It is known from previous literature that specific chemokines and chemokine receptors have been implicated in inflammatory demyelinating diseases of the central nervous system (CNS), including multiple sclerosis (MS) and experimental autoimmune encephalomyelitis (EAE). Both CCR1 and CCR5 are highly expressed in the CNS during the active phase of EAE. Met-CCL5 blocks both receptors, but it was demonstrated that Met-CCL5 treatment did not reduce CNS cellular infiltrates or up-regulation of CCR1 and CCR5 in affected CNS tissues. The variant might reduce a chemokine-mediated component of CNS macrophage and microglial activation, thus diminishing axonal pathology and neurological disability during chronic EAE, but it doesn't affect the trafficking of inflammatory hematogenous cells [11].

Based on recent studies confirming the role of CCL5 in the development and progression of EAE and its up-regulation in murine EAE, the increased potency of [^44^AANA^47^]-CCL5 over Met-CCL5 could be attributed to its proposed mode of action in that it inhibits oligomerization, a property of CCL5, and Met-CCL5, that has been shown to induce certain activation events [2].

Additionally, a recent study has implicated a key role for CCL5 in viral-induced demyelination [22]. The significant reduction in clinical score and delayed onset of disease symptoms in mice treated with [^44^AANA^47^]-CCL5 validate an approach involving disruption of chemokine-GAG interactions as a therapeutic anti-inflammatory strategy [2].

In summary, with respect to other inflammatory disorders, we demonstrated that antagonism of CCL5 showed therapeutic efficacy in models of contact skin reaction, both in a model of innate immunity (ICD) as well as in models of T-cell mediated skin pathology (CHS). Then we also demonstrated that the amino-terminal modified Met-CCL5 gave a pharmacological dose related effect and it was able to modulate innate immune response as well as T-cell driven immune responses in ICD and CHS mouse models. Data are also supported by the immunohistochemical investigations of keratinocyte pathway and kinetics of inflammatory cells proliferation (data not shown).

In conclusion, this study has been valuable in determining whether blocking the CCL5 receptor(s), involved in the recruitment of leukocytes to inflammatory sites, relieves chronic inflammation. These results demonstrate that blocking the receptor or the ligand are both effective strategies to inhibit skin inflammation.
